# A hybrid deep learning and large language model framework for MACE risk prediction and evidence-based clinical recommendations

**DOI:** 10.3389/fcvm.2026.1774938

**Published:** 2026-08-13

**Authors:** Saba Arif, Sang Hyeok Son, Jong Yun Lee

**Affiliations:** Department of Computer Science, Chungbuk National University, Cheongju, Republic of Korea

**Keywords:** cardiovascular risk prediction, deep learning, health recommendation guideline, large language models (LLM), MACE

## Abstract

**Background:**

Major adverse cardiovascular events (MACE) remain a leading cause of global morbidity and mortality, necessitating accurate risk prediction and actionable prevention strategies. This study proposes a two-fold framework integrating deep learning and large language models (LLMs) to predict MACE risk and generate personalized, guideline-based recommendations.

**Methods:**

Emergency medical record (EMR) data from Chungbuk National University (CBNU) Hospital were preprocessed and split into training and test sets, with class imbalance addressed using SMOTETomek. A one-dimensional convolutional neural network (1D-CNN) was developed to predict individual MACE risk from clinical features. To translate predictions into practice, a retrieval-augmented generation (RAG) pipeline with prompt engineering was implemented using LLM Model (MISTRAL AI), grounded in WHO and AHA cardiovascular prevention guidelines. Predictive performance was evaluated using accuracy, precision, recall, and F1-score. The recommendation system was assessed using a rubric-based LLM judge evaluating guideline fidelity, clinical safety, dietary plans, lifestyle/exercise guidance, and OTC medication caution.

**Results:**

The proposed1D-CNN achieved strong performance, with overall accuracy of 0.97. For the MACE class, precision and recall reached 1.00, with an F1-score of 0.98. The recommendation system demonstrated high guideline fidelity (5/5 for both WHO and AHA), strong dietary and lifestyle guidance (5/5), good clinical safety (4/5), and moderate OTC medication caution (3/5).

**Conclusion:**

The proposed framework effectively combines accurate MACE risk prediction with interpretable, evidence-based preventive recommendations delivered via a web interface. This integrated approach supports clinical decision-making and personalized cardiovascular risk management.

## 1 Introduction

Cardiovascular diseases (CVD) continue to have a significant impact on global health, causing millions of deaths each year and creating substantial socioeconomic challenges (1–3). Major adverse cardiovascular events (MACE) such as myocardial infarction, angina, and heart failure—are key clinical outcomes that require accurate risk assessment and tailored, proactive interventions. Traditional risk assessment tools, such as the Framingham Risk Score (4) and the ASCVD Risk Estimator (5), are widely used but often fail to account for the complex and changing interactions of clinical, demographic, and temporal risk factors, especially in diverse or high-risk groups. This challenge has led to an increasing adoption of artificial intelligence (AI) methods that use detailed datasets to improve prediction accuracy and support personalized treatment approaches.

Recent advancements in machine learning (ML) have significantly improved cardiovascular risk prediction (6–8). For instance, Huang et al. (9) introduced novel data sources, including lifestyle factors and continuous blood pressure monitoring, and trained an ensemble ML algorithm for cardiovascular risk prediction. Similarly, Adedayo et al. (10) developed a heart disease prediction system using seven ML models while addressing dataset imbalance. Yashudas et al. (11) proposed DEEP-CARDIO, an IoT-enabled framework that collects physiological data via biosensors and applies a BiGRU-attention model to diagnose multiple cardiovascular conditions and provide treatment-related outputs. Majula et al. (12) introduced MDensNet201-IDRSRNet, a hybrid deep learning model designed to enhance predictive accuracy, while Hira et al. (2) developed ensemble and blending models (EnsCVDD-Net and BlCVDD-Net) for improved heart disease prediction.

Although these studies demonstrate substantial progress in predictive modeling, their primary focus remains on improving classification performance. They do not provide systematically validated, guideline-based preventive recommendations aligned with established authorities such as the World Health Organization (WHO) and the American Heart Association (AHA). Moreover, existing strategies have not explicitly addressed the alignment and safety of large language model (LLM)-generated recommendations with medical guidelines. Given that major adverse cardiovascular events (MACE) represent a highly safety-critical condition, ensuring that risk predictions are accompanied by clinically sound, guideline-adherent recommendations is essential.

Despite significant progress in machine learning–based CVD prediction, several critical challenges remain, particularly in translating risk estimates into safe, evidence-based clinical action. First, most existing predictive models focus primarily on improving classification accuracy, yet they concluded their work at risk estimation and do not convert predictions into practical, patient-specific interventions grounded in established medical guidelines. As a result, the clinical utility of these systems remains limited. Second, although LLMs have recently shown promise in generating medical text and recommendations, their use in producing structured, guideline-specific cardiovascular prevention advice remains largely unexplored. Importantly, none of the previously proposed approaches incorporates a systematic mechanism to ensure that generated recommendations are aligned with authoritative regulatory standards such as those issued by the WHO and AHA. For example, while Yashudas et al. (11) developed a recommendation-oriented framework, it does not explicitly ensure adherence to WHO or AHA guidelines. Given that MACE and other cardiovascular conditions represent safety-critical health outcomes, both risk prediction and subsequent recommendations must strictly comply with validated clinical guidelines to ensure reliability, safety, and real-world applicability.

To address the identified gaps in cardiovascular risk prediction and guideline-based intervention, this study is motivated by the following specific objectives. First, to develop a robust and high-performing deep learning model based on a one-dimensional convolutional neural network (1D-CNN) capable of accurately predicting major adverse cardiovascular event (MACE) risk using structured EMR data. Second, to design and integrate a retrieval-augmented generation (RAG)–based Mistral large language model (LLM) that translates predicted risk scores into personalized, guideline-constrained lifestyle and preventive recommendations aligned explicitly with WHO and AHA cardiovascular prevention standards. Third, to ensure that the proposed prediction–recommendation framework remains generalizable and applicable across diverse clinical profiles, enhancing its relevance for real-world healthcare settings. Finally, to establish a unified, safety-aware decision-support system that combines data-driven risk stratification with evidence-based recommendations, thereby strengthening human–AI collaboration and supporting proactive cardiovascular risk reduction and patient-centered care.

Our methodology is designed to maintain relevance across a wide range of clinical profiles, mirroring the diversity of patient populations encountered in daily practice. This hybrid technique not only achieves exceptional predictive accuracy but also enhances human-AI collaboration in medicine by translating complex data into straightforward, actionable insights for clinicians and patients. As digital health continues to evolve rapidly, our framework offers a promising approach to reducing MACE rates, addressing healthcare disparities, and enabling individuals to manage their cardiovascular health actively. The highlights of this article are as follows:

1. This work proposes a two-fold hybrid framework for accurate MACE risk prediction and guideline-based recommendations.
2. We develop a 1D-CNN model that predicts MACE risk with improved performance compared to existing methods.
3. Prompt-engineered RAG ensures that all recommendations strictly follow WHO, and AHA, medical guidelines.
4. By extensively evaluating the alignment of the generated recommendations with WHO and AHA guidelines, we demonstrated strong guideline adherence and consistency within the proposed framework.
5. SHAP-based explanations provide patient-specific insights, enhancing model transparency and clinical trust.

## 2 Related work

This section reviews machine learning models developed for cardiovascular disease prediction and provides a thorough analysis of recent progress in the field. It emphasizes key methods, important advancements, and major findings, offering a clear overview of how ML and DL techniques have been used to enhance CVD risk assessment and prediction.

### 2.1 Machine learning-based heart disease prediction

Heart disease is one of the leading causes of mortality worldwide (13, 14), driving extensive research into computational prediction models. Traditional machine learning (ML) algorithms, such as Logistic Regression (LR), Support Vector Machines (SVM), Decision Trees (DT), Random Forests (RF), k-Nearest Neighbors (KNN), and Naïve Bayes (NB), have been extensively utilized in the analysis of clinical datasets, such as the UCI Heart Disease dataset and the Cleveland Clinic dataset (15–17). These models achieved promising accuracy and interpretability due to their statistical transparency and lower computational cost. In a study, Sadr et al. (18) proposed a hybrid approach to predicting cardiovascular disease by combining traditional machine learning classifiers, like K-Nearest Neighbor (KNN) and Extreme Gradient Boosting (XGB), with deep learning models such as Convolutional Neural Networks (CNN) and Long Short-Term Memory (LSTM) networks. Their system was trained on public heart disease datasets and a local hospital dataset, achieving improved prediction accuracy over single-model approaches. This study highlights the benefits of integrating various learning techniques for more effective multi-class CVD risk assessment. Ameen et al. (19) conducted a systematic review of 82 studies that used ML and DL methods for classifying cardiovascular disease through ECG and PCG signals. The review highlighted algorithms like Support Vector Machines, Random Forest, and Convolutional Neural Networks, with many methods achieving over 90% accuracy. However, it also pointed out challenges such as reliance on small datasets, overfitting risks, and limited clinical validation, emphasizing the need for models that ensure both accuracy and generalizability across clinical settings.

Further, Anjum et al. (20) conducted a study predicting myocardial infarction using a heart failure dataset. They evaluated six machine-learning algorithms: Logistic Regression, Support Vector Machine, Decision Tree, Bagging, XGBoost, and LightGBM, using metrics like accuracy, precision, recall, F1-score, and AUC. Results showed that gradient-boosted ensembles (XGBoost, LightGBM) outperformed linear and tree models, while Logistic Regression acted as a practical reference model. The study emphasizes the importance of systematic model comparison and metric-driven evaluations in clinical cardiovascular decision support. A recent study (21) introduced an Attention-Based Cross-Modal (ABCM) transfer learning framework to enhance CVD prediction by integrating clinical, imaging, and genetic data. This framework employs an attention mechanism to focus on the most relevant features across different data sources, improving prediction accuracy and interpretability over traditional models. Results showed superior performance compared to conventional machine learning methods in metrics like accuracy, precision, recall, and AUC. The framework's robustness was validated through Monte Carlo simulations, ANOVA testing, and ablation studies, highlighting the effectiveness of cross-modal attention in CVD risk assessment. Also, Kumar et al. (22) applied KNN, Logistic Regression, Random Forest, and XGBoost on the Cleveland Heart Disease dataset and a private dataset for early heart disease prediction. They used SMOTE for data imbalance and SHAP for explainability, with XGBoost achieving an accuracy of 97.57%, showcasing its potential for real-time diagnostics. Despite these advancements, the majority of prior work focuses solely on improving prediction accuracy, without translating risk estimates into personalized, actionable health guidance for patients.

### 2.2 Recommendation systems in health care

In addition to predictive modeling, recommendation systems (RS) have gained attention as valuable decision-support tools for both clinicians and patients (23–25). Recent advancements have introduced data-driven recommender models that utilize machine learning to offer personalized guidance on diet, exercise, and lifestyle changes. Dogan et al. (26) developed a utility-based machine learning model that recommends personalized lifestyle changes such as diet, exercise, and smoking cessation to help prevent cardiovascular disease. The model ranks these lifestyle options based on the predicted health benefits for each individual, focusing primarily on disease prevention rather than diagnostic predictions. Similarly, Martínez-Rodrigo et al. (27) developed a web-based recommendation framework aimed at promoting healthy vascular aging. This system provides personalized advice regarding nutrition and physical activity based on individual user data. However, it does not include machine learning for disease diagnosis. In another study (28), Sun et al. conducted a scoping review of 51 studies on health recommender systems, focusing on application areas, recommendation techniques, and evaluation strategies. The review highlighted a growing interest in hybrid approaches, noting that most systems are geared toward general wellness or lifestyle management. It also pointed out that there are relatively few applications that integrate diagnostic prediction with personalized health recommendations. In study (29) López-Barreiro et al. conducted a systematic review of AI-driven recommender systems designed to promote healthy habits and support active aging. The study analyzed various system architectures and use cases, focusing on the role of AI in providing personalized recommendations aimed at enhancing physical, cognitive, and social well-being. The review indicates notable advancements in health-focused recommender technologies; however, it also notes that applications targeting specific diseases or integrating predictive models with recommendations are relatively limited.

Medication-oriented recommender systems have also gained attention in recent years. Mai et al. (30) developed a hybrid clinical decision support system for antihypertensive drug recommendations, merging data-driven learning with clinical rules. By using patient demographic and clinical data, the model effectively ranked medications according to treatment guidelines and aligned well with expert recommendations. While the study highlighted the potential of machine learning in optimizing hypertension therapy, it focused solely on treatment selection without incorporating predictive diagnostics or personalized AI adaptations. Similarly, Afanasieva et al. in article (23) developed a knowledge-based recommendation algorithm aimed at supporting the self-management of cardiovascular risk factors at home. This algorithm combines rule-based reasoning with natural-language explanations to improve patient understanding and engagement. However, their primary focus is on treatment optimization rather than the integration of predictive diagnosis with adaptive recommendations.

Overall, previous research has advanced heart disease prediction and health recommender systems, but few have combined them in a unified, interpretable framework. To bridge this gap, our study presents a machine learning–based model that integrates explainable analytics and personalized guidance to improve cardiovascular risk assessment and clinical decision support.

## 3 Proposed approach

### 3.1 Experimental setup

**Figure 1:**
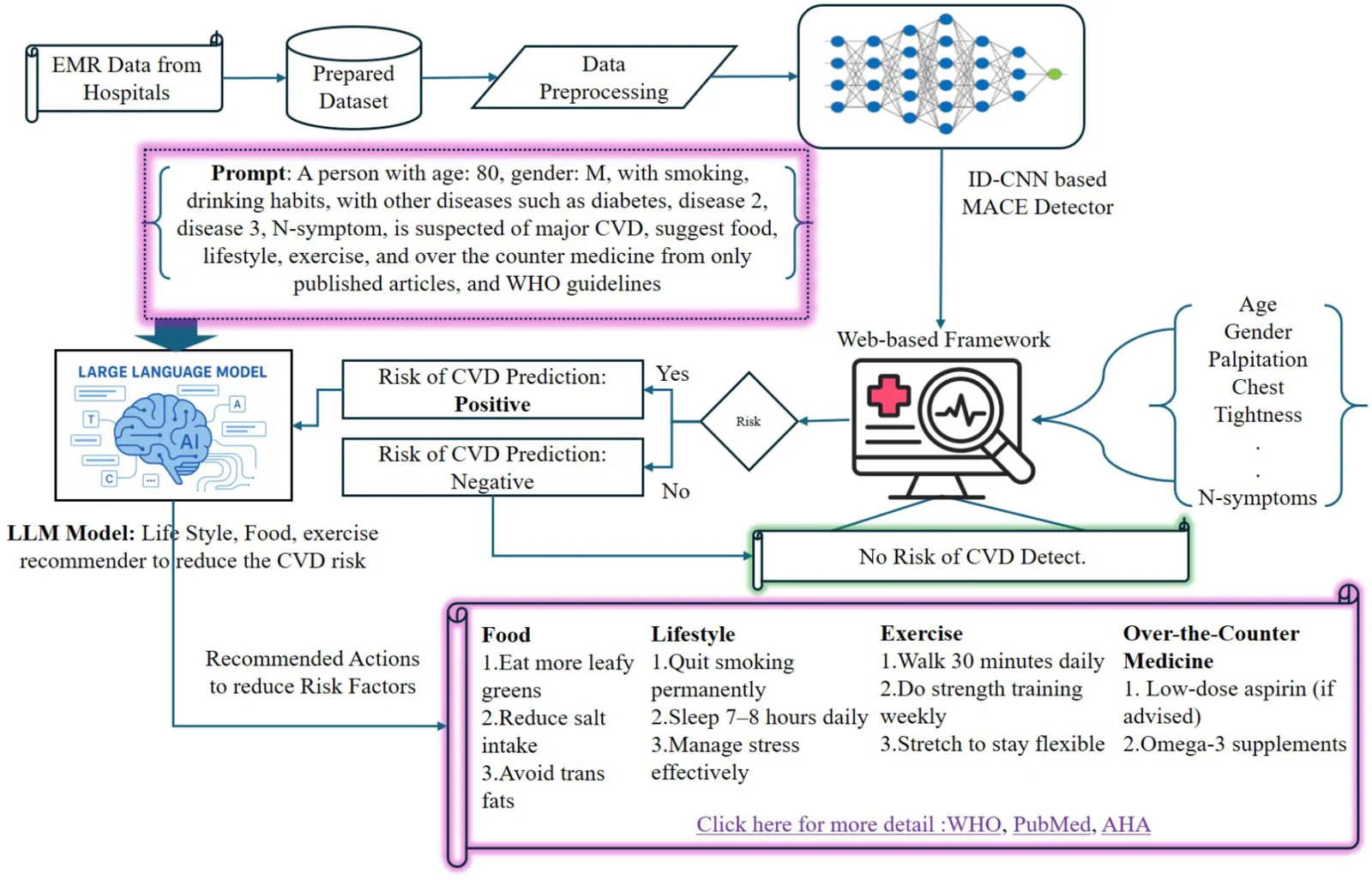
A hybrid deep learning and large language model framework for MACE risk prediction and evidence-based clinical recommendations.

The overall experimental workflow of the proposed approach for MACE risk prediction and evidence-based clinical recommendations is presented in Figure 1. First, our experimental data is extracted from the Chungbuk National University (CBNU) Hospital's emergency medical record (EMR) dataset and preprocessed the extracted data, i.e., handling irrelevant variables and missing values, encoding some categorical variables. Second, the experimental dataset is split into training (80%) and test data (20%) while considering the percentage of each class for further experiment. However, there is a class imbalance problem of experimental data after extraction. Therefore, we consider SMOTETomek hybrid sampling approach to balance the dataset. SMOTETomek is a hybrid resampling technique used to address class imbalance in machine learning datasets. It combines SMOTE, which creates synthetic minority class samples, with Tomek Links, which remove borderline or noisy majority samples. By applying oversampling and cleaning simultaneously, it helps produce a more balanced and better-separated dataset. This improves the quality of training data and reduces the risk of overfitting. Overall, SMOTETomek enhances model performance, especially in classification tasks involving rare classes. Third, we develop a 1D convolutional neural network (1D-CNN) prediction model to predict the MACE occurrences in patients. We then apply carefully designed prompt engineering and RAG to MISTRAL AI for recommendation of clinical actions to reduce the risk MACE by strictly following the WHO and AHA guidelines. Lastly, we evaluate the performance of the 1D-CNN-based MACE predictor and LLM-based recommendations systems using the standard metrics.

### 3.2 Data source and data extraction

For the experiment, we use the CBNU's hospital EMR dataset, which contains the data medical records of all departments including cardiology. The raw dataset after excluding other departments contains 221,490 patients’ medical records from July 2020 to February 2023.

The CBNU's hospital EMR dataset includes numerous irrelevant variables and plenty of missing values, so we extract the raw dataset before developing the prediction model. During the preprocessing we first eliminate the irrelevant variables which have no impact on the prediction of MACE occurrences (31). In this paper, our target values are defined as ‘No_MACE’ and ‘MACE’, where the MACE was defined as in-hospital occurrences of myocardial infarction, unstable angina requiring hospitalization, or hospitalization for heart failure documented after the index admission and before discharge. During the preprocessing phase, the values of all categorical variables are also converted into the integer values. For example, the value ‘Y/N′ representing the “YES/NO” is converted into ‘1/0′. The applied variables extracted from the CBNU Hospital's EMR dataset are shown in Table 1.

**Table 1:** The applied variables from the CBNU hospital's EMR dataset.

Variable type	Clinical variables
Categorical (14)	Gender (male/female/other) Chest Pain (present/absent) Both Leg Edema (yes/no) Both Flank Pain (yes/no) Chest discomfort (yes/no) Dyspnea (yes/no) Dizziness (yes/no) Syncope (yes/no) Palpitation (yes/no) Leg Swelling (yes/no) Edema Leg (yes/no) Flank Pain (yes/no) Shortness of Breath (yes/no) Chest Tightness (yes/no)
Continuous (4)	BMI (kg/m^2^) Age (years) Temperature ( °C) Breathing (rate per min) Pulse (beats per minute) Systolic Blood Pressure (mmHg) Diastolic Blood Pressure (mmHg)

**Figure 2:**
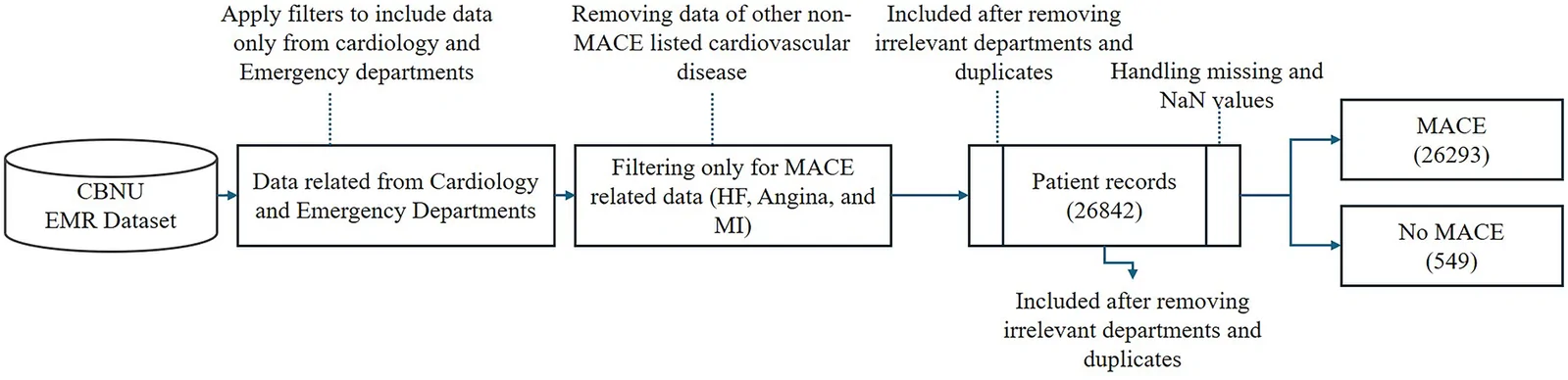
The process of data extraction.

**Figure 3:**
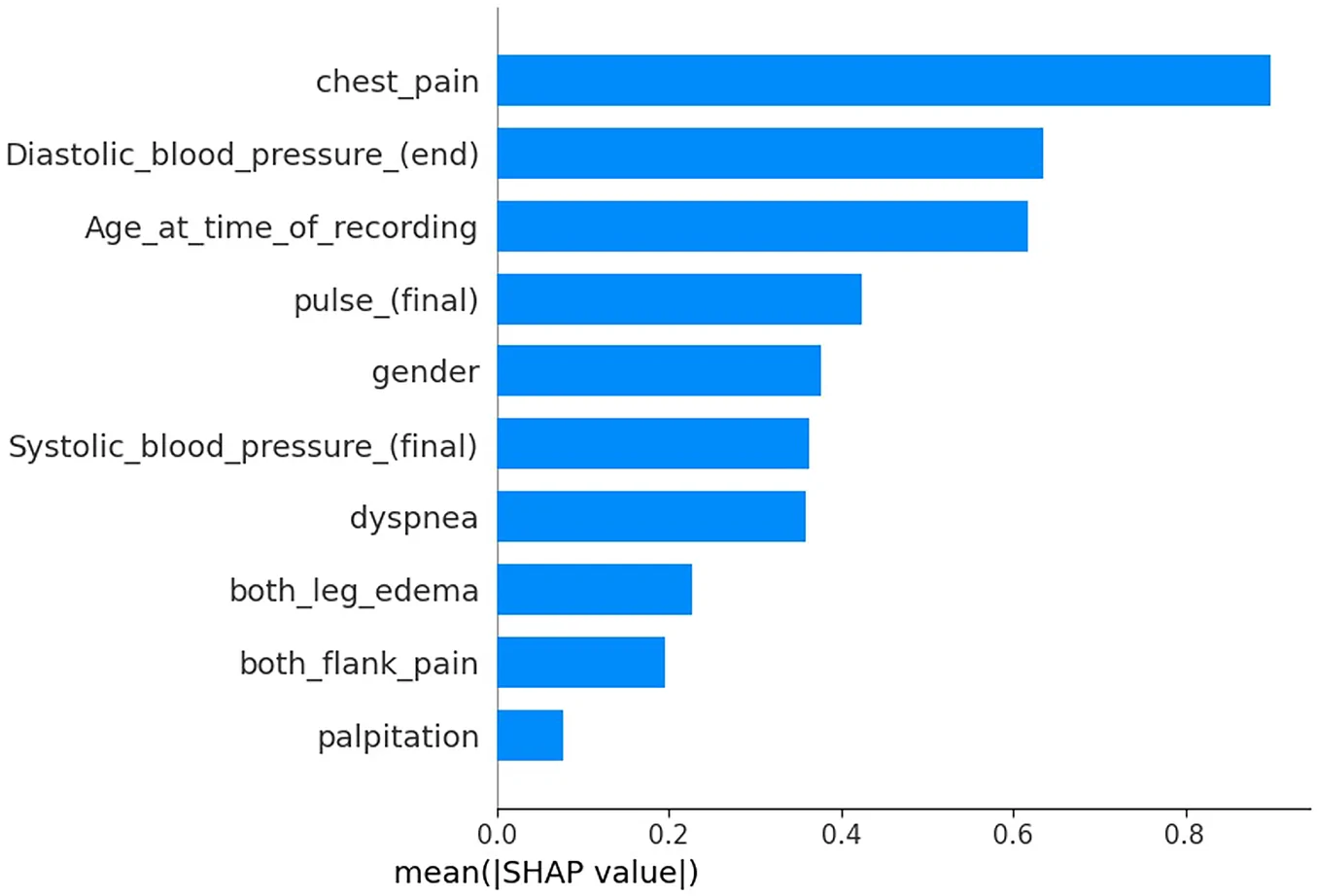
Top 10 important features among the selected features.

From the experimental dataset, we then we exclude the patients’ records that does not belong to emergency and cardiology departments. After the first filter, we apply second filters to remove the patient records that does not belong the list of cardiovascular diseases included in MACE. The data extraction process is illustrated in Figure 2, where the final dataset includes the MACE records of 26,293 (97.95%) and No_MACE 549 (2.04%). However, there are still amounts of missing values in the final dataset. To deal with the missing values, we apply two different types of imputers such as median and most frequent. The median imputer is effective for numerical features because it is robust to outliers. By replacing missing entries with the median of the column, it helps maintain the overall distribution of the data without being influenced by extreme values. On the other hand, the most frequent imputer is suitable for categorical features where numerical measures like mean or median do not apply. It fills in missing values using the most commonly occurring category, preserving the dominant pattern within the feature. Together, these imputers ensure that the dataset remains consistent and complete before modeling. The feature importance is calculated based SHAP value. SHAP-based feature importance measures how much each feature contributes to a model's predictions by using Shapley values from cooperative game theory. It provides a consistent and interpretable way to understand the impact of each feature by evaluating how predictions change when the feature is included or excluded. Unlike traditional importance measures, SHAP values offer both global and local interpretability, showing overall feature influence as well as contribution for individual predictions. Numerical columns are also imputed with simple imputer technique using the median strategy and the categorical columns were imputed using the “most frequent” strategy i.e., mode-based imputation. Among the selected features, chest pain, Diastolic Blood pressure and age remain the top features that influence the final predictions. Figure 3 presents the top 10 important features that influence the final predictions. More detail about the features and their influence on final predictions will be discussed in results section.

The index date was defined as the date of the patient's first hospital admission for cardiovascular disease in the emergency or cardiology department during the study period (July 2020–February 2023). The prediction window was limited to the index hospitalization, and patients were followed from admission to discharge. Only MACE events occurring after admission and before discharge were considered. In this study, MACE was defined as a composite endpoint consisting of myocardial infarction, unstable angina requiring hospitalization, and hospitalization for heart failure, based on the outcomes available in the CBNU Hospital EMR dataset. Events recorded at the time of admission were included only if documentation indicated that they occurred during hospitalization. We included adults (≥13 years) with a first recorded cardiovascular admission during the study period and available baseline admission data. We excluded patients with MACE that could not be temporally distinguished from admission, missing outcome data, or repeated admissions (only the first admission per patient was retained).

### 3.3 Proposed 1D-CNN-based MACE prediction model

In this study, we propose a one-dimensional convolutional neural network (CNN) to predict the occurrence of MACE using the tabular data. Traditional approaches to handling tabular data, such as multilayer perceptron (MLPs), use fully connected layers that treat each feature independently. This assumption leads to uniform relevance among features and fails to explicitly capture local feature dependencies. In contrast, our proposed CNN-based model takes advantage of the ordered representation of tabular features by interpreting the feature vector as a one-dimensional signal. By applying convolutional filters across this feature space, the model can detect localized interactions and hierarchical representations that are often overlooked by standard MLP architectures.

The input to the network is a preprocessed feature vector x∈R^d reshaped into a one-dimensional sequence of length d with a single channel. The convolutional backbone of the network comprises three convolutional layers with a kernel size 3 and padding to preserve sequence length. The first layer projects the input into 64 channels, the second layer expands this representation to 128 channels, and the third layer maintains the dimensionality at 128 channels. Each convolutional operation is followed by batch normalization, rectified linear unit (ReLU) activation, and dropout regularization with *p* = 0.1. Mathematically, each convolutional layer can be described as shown in equation (1):

$$h_{i,j}=\sigma \left(\overset{C_{in}}{\underset{C=1}{\sum }}\overset{K}{\underset{K=1}{\sum }}W_{j,c,k}x_{i+k-1,c}+b_{j}\right)$$ (1)

Where $W_{\;j,c,k}$ are the kernel weights, $b_{j}$ is learnable bias term, K=3 is the kernel size and σ(.) denotes the ReLU nonlinearity. Batch normalization ensures numerical stability and accelerates convergence by re-centering and re-scaling activations, while dropout reduces overfitting by randomly deactivating units during training, as expressed in equation (2).

$$o=W_{2}\;\sigma (W_{1}z+b_{1})+b_{2}$$ (2)

To achieve dimensionality invariance with respect to the sequence length, adaptive max pooling is applied after the final convolutional layer. This operation reduces each feature map to its maximum activation value, resulting in a fixed-length vector $z\in R^{128}$, independent of the input dimensionality. The pooled representation is then processed by a two-layer fully connected classifier. The first dense layer maps the feature representation from 128 to 64 dimensions, followed by ReLU activation and dropout, while the second dense layer produces a scalar logit o∈R, representing the unnormalized evidence for the positive class. As shown in equation (3), the transformation can be expressed as:

$$\hat{y}=\;\sigma (o)=\frac{1}{1+e^{-o}}$$ (3)

Where $W_{1}\in R^{64\times 128}$ and $W_{2}\in R^{1\times 64}$ are learnable weight matrices with corresponding biases $b_{1}$ ​ and $b_{2}$ ​. At inference, the scalar logit is passed through a sigmoid activation, which maps the output to a probability in the interval [0,1]. A threshold of 0.5 is applied to generate the final binary prediction.

Compared to MLPs, which typically consist of stacked fully connected layers operating on flat feature vectors, this architecture introduces three key innovations. First, the use of convolutional layers enforces a local connectivity pattern, enabling the model to exploit short-range feature dependencies rather than assuming full independence across dimensions. Second, the hierarchical channel expansion (from 1 to 128 channels) allows the model to learn increasingly abstract feature representations while retaining fine-grained local information. Finally, the application of adaptive max pooling guarantees a fixed-length representation, introducing invariance to the number of features and reducing the risk of overfitting to specific feature indices.

The proposed 1D-CNN model for predicting the occurrence of major adverse cardiac events (MACE) consists of a total of 12 learnable layers, including 3 convolutional layers, 3 batch normalization layers, and 2 fully connected layers, along with their associated dropout and pooling layers. The architecture features a hierarchical expansion of channels from 1 to 128 in the convolutional stack, followed by dimensionality reduction from 128 channels to 1 in the fully connected block. This design provides both expressive power and compact decision-making capability. In comparison to multi-layer perceptron (MLPs), which typically only include fully connected transformations, this architecture incorporates local feature extraction, hierarchical abstraction, and regularization mechanisms, making it particularly well-suited for high-dimensional tabular classification.

### 3.4 Proposed evidence-based clinical recommendation system

This paper also proposes a recommendation system for cardiovascular disease (CVD) prevention based on the Mistral (32) AI large language model (LLM). The system leverages a quantized version of the Mistral-7B-Instruct model (Q4_K_M), deployed using the ‘ctransformers’ library (33) to balance computational efficiency and semantic fidelity. The objective was to generate personalized recommendations for patients at elevated cardiovascular risk, strictly aligned with World Health Organization (WHO) and American Heart Association (AHA) guidelines for MACE (Major Adverse Cardiovascular Events) risk reduction.

Our recommendation system integrates Retrieval-Augmented Generation (RAG) (34) and prompt engineering (35). The RAG component ensures that outputs are constrained by evidence-based guidelines, while prompt engineering provides structural conditioning of the model's responses. Specifically, the prompt encodes a quantified patient risk scenario [Pr(CVD) = 0.6] and explicitly instructs the model to deliver structured recommendations across diet, lifestyle, exercise, and over-the-counter medication.

**Figure 4:**
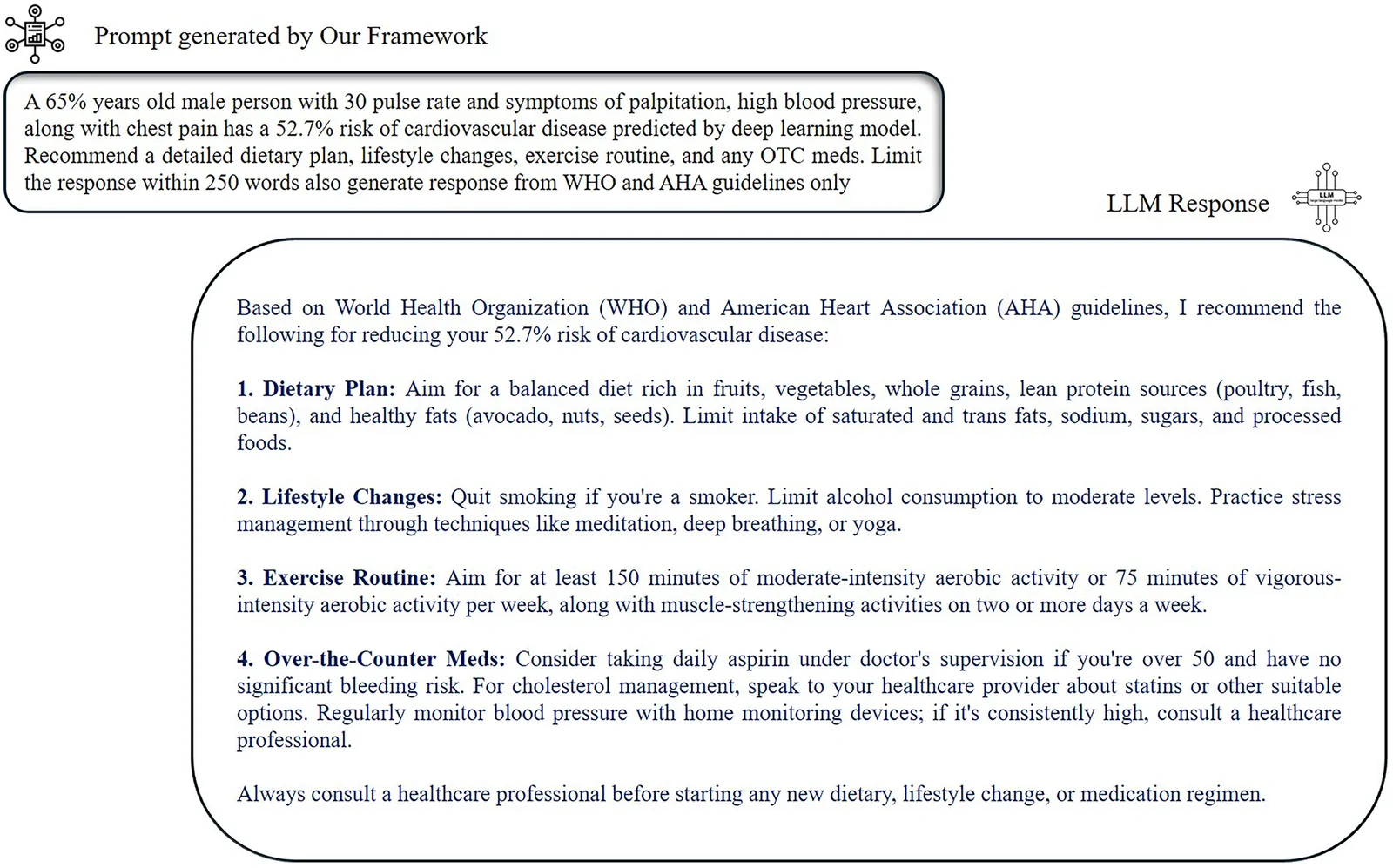
Clinical recommendation generated by recommendation system under wHO and AH guidelines.

**Figure 5:**
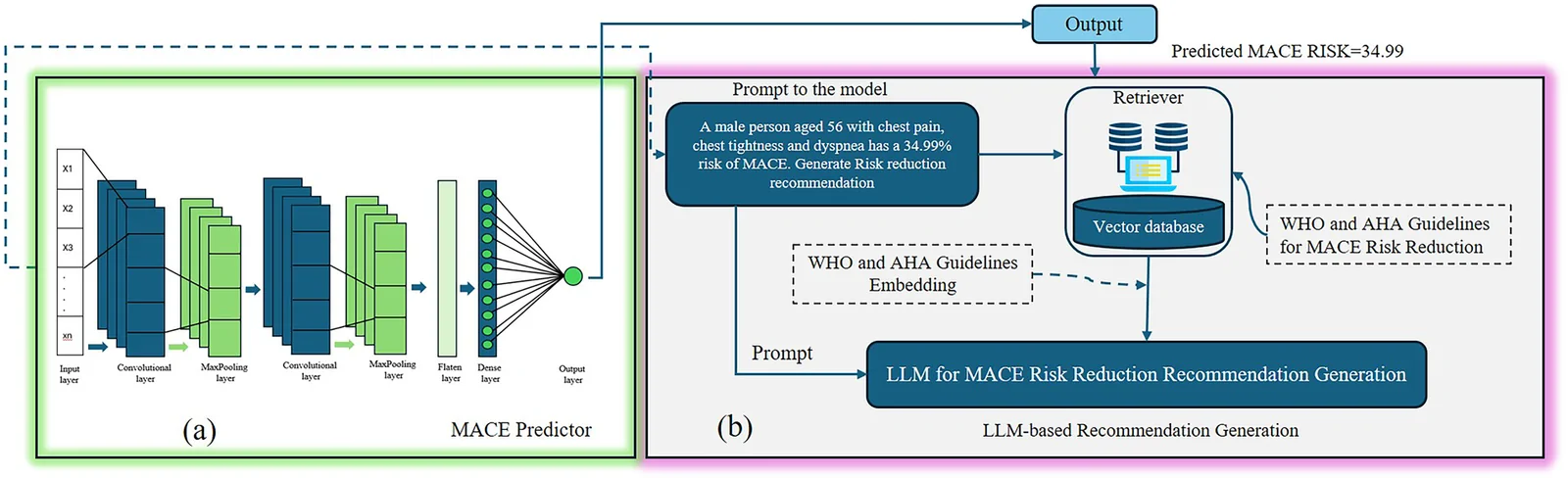
Internal architecture of 1D-CNN-MACE predictor and LLM-based recommendation system. **(a)** shows the internal architecture of predictor, and **(b)** shows the recommendation system.

Formally, the system generates a recommendation R by solving, as shown in equation (4):

$$R=\;arg\underset{y\in Y}{\max}p(y|\;P,\theta ,D)$$ (4)

where P denotes the engineered instruction prompt, *θ* the pretrained Mistral.AI model parameters, and D the retrieved domain-specific guidelines. The inclusion of D enforces guideline-constrained text generation, mitigating hallucination risks. An illustrative example of how the recommendation system works presented in Figure 4. It provides an illustrative example demonstrating how the recommendation system works in practice. It shows how the predicted MACE risk level triggers the retrieval and generation of personalized, guideline-based prevention advice using the RAG-enabled LLM. Figure 5 presents the internal architecture of the entire framework. This shows the two-part workflow of the proposed system. First, the 1D-CNN predicts MACE risk using EMR-derived risk inputs. Second, this predicted risk is passed to the LLM module, which uses prompt engineering and RAG to retrieve guideline-based information and generate personalized prevention recommendations.

### 3.5 Statistical analysis

For the statistical analysis of the experimental data and feature importance assessment, we employed both the Chi-square test (36) and the independent t-test (37), depending on the type of variable. Specifically, the Chi-square test was used to evaluate the association between categorical features and the MACE outcome. This test allowed us to determine whether the distribution of categorical variables differed significantly between the MACE and non-MACE groups. The importance of categorical features was determined based on the corresponding *p*-values obtained from the Chi-square analysis.

For continuous variables, we applied the independent (two-sample) t-test to assess whether there were statistically significant differences in mean values between the two groups. The *p*-values derived from the t-test were used to determine the statistical significance and relative importance of continuous features.

Categorical variables are presented as frequencies and percentages (n, %), while continuous variables are reported as mean ± standard deviation (SD). A significance threshold (e.g., *p* < 0.05) was used to identify statistically meaningful features for subsequent modeling. These statistical analyses ensured a systematic and transparent feature selection process prior to model development.

### 3.6 Evaluation metrics

To evaluate the performance of our MACE occurrence predictor model, we use accuracy, precision, recall, and F1-score. These evaluation metrics are defined in Equations 5–8, where TP, TN, FP, and FN are the means the true positive, true negative, false positive, and false negative, respectively.

$$Accuracy=\frac{TP+TN}{TP+TN+FP+FN}$$ (5)

$$Precision=\frac{TP}{TP+FP}$$ (6)

$$Recall=\frac{TP}{TP+FN}$$ (7)

$$F1-score=\frac{2\times (recall\times precision)}{recall\times precision}$$ (8)

### 3.7 Implementation details

#### 3.7.1 Hardware and software specifications

All experiments were implemented on a system with Intel Core i7 2.70 GHz, and 16GB random access memory (RAM) using the Microsoft Windows 10 operating system. We used the PyCharm, and Jupyter Notebook to develop the model architecture. The trained model was deployed for web application using the Flask library. To carry out the experiments, the Python (Version 3.10) fancyimpute library to fill in the missing values, the imbalancedlearn library to solve the class imbalance problem. We also used other libraries such as the scikit-learn package and XGBoost library to develop the machine learning model for comparative analysis. The models were developed using Python, while the web application interface was designed and implemented using HTML, JavaScript, and CSS.

The MACE prediction model was first trained using the CBNU Hospital dataset and subsequently deployed on a local machine for inference. For recommendation generation, we utilized the pre-trained Mistral model. The system operates by receiving user input through the web-based interface; the 1D-CNN model then predicts the MACE risk score. This risk score, along with user-reported active symptoms and a structured prompt, is provided to the Mistral model, which generates personalized recommendations aligned with WHO and AHA guidelines.

To evaluate the alignment of the generated recommendations with WHO and AHA standards, we employed Llama 3.1 8B via Ollama as a rubric-based LLM judge. The prompts, predicted MACE risk scores, and relevant WHO and AHA guideline content were fed into Llama 3.1 8B, which produced structured evaluation scores assessing guideline adherence and recommendation quality.

#### 3.7.2 Hardware and software specifications

Since the Mistral model was used in its pretrained form without fine-tuning, tunable parameters were limited to prompt design and inference-time generation settings. We evaluated multiple prompt templates, including zero-shot and few-shot variants, and implemented an explicit constraint-based instruction enforcing strict adherence to WHO and AHA guidelines. During inference, generation hyperparameters were tuned within controlled ranges (temperature: 0.0–0.7; selected 0.2, top_p: 0.6–0.95; selected 0.9, top_k: 0–100; selected 40, max_tokens: 200–800; selected 360, repetition_penalty: 1.0–1.5; selected 1.2). Lower temperature values were chosen to improve determinism and clinical safety. For rubric-based evaluation using Llama 3.1 (8B), temperature was set to 0.0 to ensure consistent scoring.

## 4 Experimental results

### 4.1 Baseline characteristics of the experimental dataset

The clinical characteristics of the study population (*n* = 26,842) demonstrated notable differences between patients with and without major adverse cardiovascular events (MACE). Overall, the mean age was 72.8 years, with MACE patients being slightly older than non-MACE patients (72.9 vs. 68.6 years, *p* < 0.001). Body mass index and body temperature showed no significant differences between groups, while pulse rate was modestly lower in the MACE group (*p* = 0.0055). Blood pressure profiles revealed that diastolic pressure was significantly higher in MACE patients (*p* = 0.0017), whereas systolic blood pressure did not differ significantly. Gender distribution also varied, with a higher proportion of males in the MACE group compared to non-MACE patients (*p* = 0.0011).

Symptom profiles indicated strong associations with adverse outcomes. Chest pain, dyspnea, and syncope were significantly more frequent in MACE patients (all *p* < 0.001). Similarly, dizziness and palpitations showed borderline associations, whereas edema and flank-related symptoms were rare but demonstrated statistical signals. Notably, dyspnea was observed in over half of the cohort (56.0%), but was less common among those with MACE compared to non-MACE patients (39.7% vs. 56.3%, *p* < 0.001). Collectively, these findings highlight that advanced age, male gender, and the presence of specific cardiovascular symptoms such as chest pain and syncope are strongly associated with the occurrence of MACE in this population. The clinical characteristics of the experimental dataset is presented in Table 2.

**Table 2:** Clinical characteristics of the experimental dataset.

Variable	All (*n* = 26,842)	MACE (Y) (*n* = 26,293)	No MACE (*N*) (*n* = 549)	*P*-value
Gender	14,456 (53.9%)	14,123 (53.7%)	333 (60.7%)	**0**.0011*
BMI	23.80 ± 4.50	23.80 ± 4.50	23.59 ± 4.47	0.4531
Age	72.83 ± 12.89	72.92 ± 12.87	68.64 ± 13.15	**0**.0000**
Body temperature	36.77 ± 1.40	36.77 ± 1.40	36.77 ± 1.62	0.9020
Breathing	19.59 ± 3.29	19.59 ± 3.30	19.39 ± 2.71	0.0919
Pulse	78.42 ± 16.80	78.46 ± 16.80	76.46 ± 16.64	**0**.0055*
Systolic blood pressure	119.52 ± 19.03	119.49 ± 19.04	120.74 ± 18.33	**0**.01164*
Diastolic blood pressure	67.60 ± 12.96	67.56 ± 12.97	69.24 ± 12.33	**0**.0017*
Chest pain	7,274 (27.1%)	7,008 (26.7%)	266 (48.5%)	**0**.0000**
Both leg edema	9 (0.0%)	9 (0.0%)	0 (0.0%)	**0**.0027*
Both flank pain	10 (0.0%)	10 (0.0%)	0 (0.0%)	**0**.0016*
Chest discomfort	83 (0.3%)	83 (0.3%)	0 (0.0%)	**0**.0000**
Dyspnea	15,033 (56.0%)	14,815 (56.3%)	218 (39.7%)	**0**.0000**
Dizziness	449 (1.7%)	432 (1.6%)	17 (3.1%)	0.0513
Syncope	249 (0.9%)	249 (0.9%)	0 (0.0%)	**0**.0000**
Palpitation	928 (3.5%)	916 (3.5%)	12 (2.2%)	**0**.0413*
Leg swelling	28 (0.1%)	28 (0.1%)	0 (0.0%)	**0**.0000**
Edema leg	141 (0.5%)	135 (0.5%)	6 (1.1%)	0.1947
Flank pain	111 (0.4%)	106 (0.4%)	5 (0.9%)	0.2136
Shortness of breath	7 (0.0%)	7 (0.0%)	0 (0.0%)	**0**.0081*
Tightness chest	25 (0.1%)	25 (0.1%)	0 (0.0%)	**0**.0000**

The single asterisk (*) with *p*-value denotes that variables are statistically signiﬁcant as *p*-value < 0.05 means that there are less than 5% chance of being wrong and double asterisk (**) with *p*-value denotes that variables are statistically high signiﬁcant as *p*-value < 0.001 means that there are less than one in a thousand chance of being wrong in MACE and No MACE groups.

Bold values indicate the statistically significant results and highlight important differences between groups.

### 4.2 Performance analysis of MACE predictor

The experimental result of the proposed MACE predictor in Table 3. The XGBoost classifier demonstrated outstanding performance across all metrics, with an accuracy of 0.97 and balanced precision and recall for both “MACE” and “No MACE” classes. Its F-scores of 0.97 for both classes reflect a highly reliable predictive capacity, nearly matching the performance of the proposed 1D-CNN model. The marginal difference lies in recall for the “MACE” class (0.94 in XGBoost vs. 1.00 in 1D-CNN), which may lead to slightly higher false negatives compared to the deep learning approach. Nonetheless, XGBoost provides a highly competitive alternative, excelling in interpretability and efficiency, though it falls short of the near-perfect classification achieved by the proposed model.

**Table 3:** Performance evaluation of proposed MACE predictor.

Model	Classes	Precision	Recall	F-score	Accuracy
XGBoost	No MACE	0.94	1.00	0.97	0.97
	MACE	1.00	0.94	0.97	
AdaBoost	No MACE	0.72	0.70	0.71	0.73
	MACE	0.74	0.76	0.75	
Logistic Regression	No MACE	0.62	0.55	0.58	0.62
	MACE	0.63	0.69	0.66	
Support Vector Machine	No MACE	0.71	0.72	0.72	0.73
	MACE	0.74	0.73	0.74	
Random Forest	No MACE	0.76	0.72	0.74	0.76
	MACE	0.76	0.79	0.78	
KNN	No MACE	0.92	1.00	0.96	0.96
	MACE	1.00	0.92	0.96	
1D-CNN Model (Proposed Model)	**No MACE**	**0**.**95**	**0**.**95**	**1**.**00**	**0**.**97**

Bold values represent the best-performing results among the compared models.

#### 4.2.1 Adaboost

In contrast, AdaBoost presented moderate classification ability, achieving an overall accuracy of 0.73. The model exhibited relatively balanced precision and recall across classes, with F-scores of 0.71 for “No MACE” and 0.75 for “MACE.” However, these results are substantially lower than those achieved by the 1D-CNN model, particularly in terms of reliability and consistency. While AdaBoost is effective at handling noisy data and reducing bias, its comparatively lower recall for “No MACE” (0.70) suggests a higher rate of false negatives, which may limit its clinical applicability in sensitive contexts. This indicates that AdaBoost struggles to capture the complex feature interactions that the CNN model successfully learns.

#### 4.2.2 Logistic regression

Logistic Regression, a baseline linear model, recorded the weakest performance among all methods, with an overall accuracy of 0.62. The F-scores of 0.58 (“No MACE”) and 0.66 (“MACE”) underscore its limited predictive power, particularly due to poor recall for the “No MACE” class (0.55). Compared to the 1D-CNN model, which achieved near-perfect classification, Logistic Regression fails to adequately model the nonlinear and high-dimensional feature space required for this task. Although the method offers interpretability and simplicity, its inferior performance highlights the inadequacy of linear decision boundaries in complex biomedical prediction problems.

#### 4.2.3 Support vector machine (SVM)

The Support Vector Machine achieved an accuracy of 0.73, with relatively balanced precision and recall across both classes (approximately 0.71–0.74). This reflects better performance than Logistic Regression but remains significantly inferior to the 1D-CNN model. While SVMs are known for their ability to handle high-dimensional data and construct optimal decision boundaries, their performance here indicates difficulty in fully capturing the intricate patterns necessary for distinguishing “MACE” from “No MACE” cases. In comparison to the CNN, which leverages hierarchical feature extraction, the SVM's limited flexibility results in lower discriminative power, particularly in terms of F-scores (max 0.74 vs. 0.98–1.00).

#### 4.2.4 Random forest

The Random Forest classifier provided a solid performance with an accuracy of 0.76, outperforming AdaBoost, Logistic Regression, and SVM. With F-scores of 0.74 (“No MACE”) and 0.78 (“MACE”), it demonstrated a balanced trade-off between precision and recall, reflecting its strength in handling nonlinear relationships and interactions among features. However, while the model surpasses several traditional approaches, it still falls short of the 1D-CNN model, which achieved near-perfect recall and precision across classes. The gap suggests that while ensemble tree methods are effective, they cannot fully replicate the representational learning advantage of deep neural networks in this context.

#### 4.2.5 K-nearest neighbours (KNN)

KNN exhibited strong predictive ability, achieving an accuracy of 0.96 with balanced performance across classes (F-scores of 0.96 each). Its recall for “No MACE” was perfect (1.00), but it slightly underperformed in identifying “MACE” cases with a recall of 0.92. Compared to the 1D-CNN, KNN performed remarkably well and emerged as one of the strongest traditional models. However, the CNN surpassed KNN by achieving perfect recall for both classes, which is crucial in clinical applications where missing a positive case (false negative) can have severe consequences. Thus, while KNN is competitive, the CNN remained superior due to its robustness and generalizability.

#### 4.2.6 1D-CNN (proposed model)

The proposed 1D-CNN model achieved excellent performance, recording an accuracy of 0.97 with nearly flawless classification across both classes. It demonstrated perfect precision and recall for the “MACE” class (1.00 each), and a perfect F-score for the “No MACE” class (1.00), indicating its superior capacity to generalize and minimize both false positives and false negatives. The CNN's strength lies in its ability to learn hierarchical feature representations directly from the input, enabling it to capture subtle and complex patterns that traditional machine learning models fail to detect. This positions the 1D-CNN as the most effective and reliable approach for the task, particularly in clinical prediction scenarios where accuracy and sensitivity are paramount.

### 4.3 Explainability of prediction results with SHAP analysis

The SHAP bar plot illustrated the average contribution of each feature to the predictive model, with higher mean SHAP values indicating stronger influence on the model's output. In this analysis, chest_pain emerged as the most impactful predictor, suggesting it plays a central role in determining the model's decisions, followed by diastolic_blood_presure_(end) and age_at_the_time_of_recording, both of which were clinically relevant indicators of cardiovascular risk. Other physiological parameters, such as pulse_(final) and systotlic_blood_pressure_(final), also contributed substantially, reflecting their established association with cardiac function. Additionally, demographic factors such as gender, alongside clinical symptoms including dyspnea, both_leg_edema, both_flank_pain, and palpitation, exhibit meaningful though relatively lower contributions. Collectively, the ranking underscored the predominance of chest_pain and blood pressure–related variables, while also highlighting the relevance of demographic and symptomatic features in shaping the model's predictions. SHAP bar plot is presented in Figure 3.

The SHAP decision plot demonstrated how individual features collectively influenced the model's prediction by visualizing their sequential contributions along the prediction pathway. In this case, age initiated the progression of effects, establishing an important demographic baseline for interpretation. This was followed by pulse, which added an early hemodynamic component that helped frame subsequent physiological impacts. The largest directional shifts were introduced by diastolic blood pressure (DSBP) and systolic blood pressure (SBP), both of which emerged as dominant contributors and reinforced the central role of blood pressure regulation in shaping cardiovascular risk. Chest pain provided additional clinically meaningful influence, reflecting a key symptomatic indicator of potential cardiac compromise.

**Figure 6:**
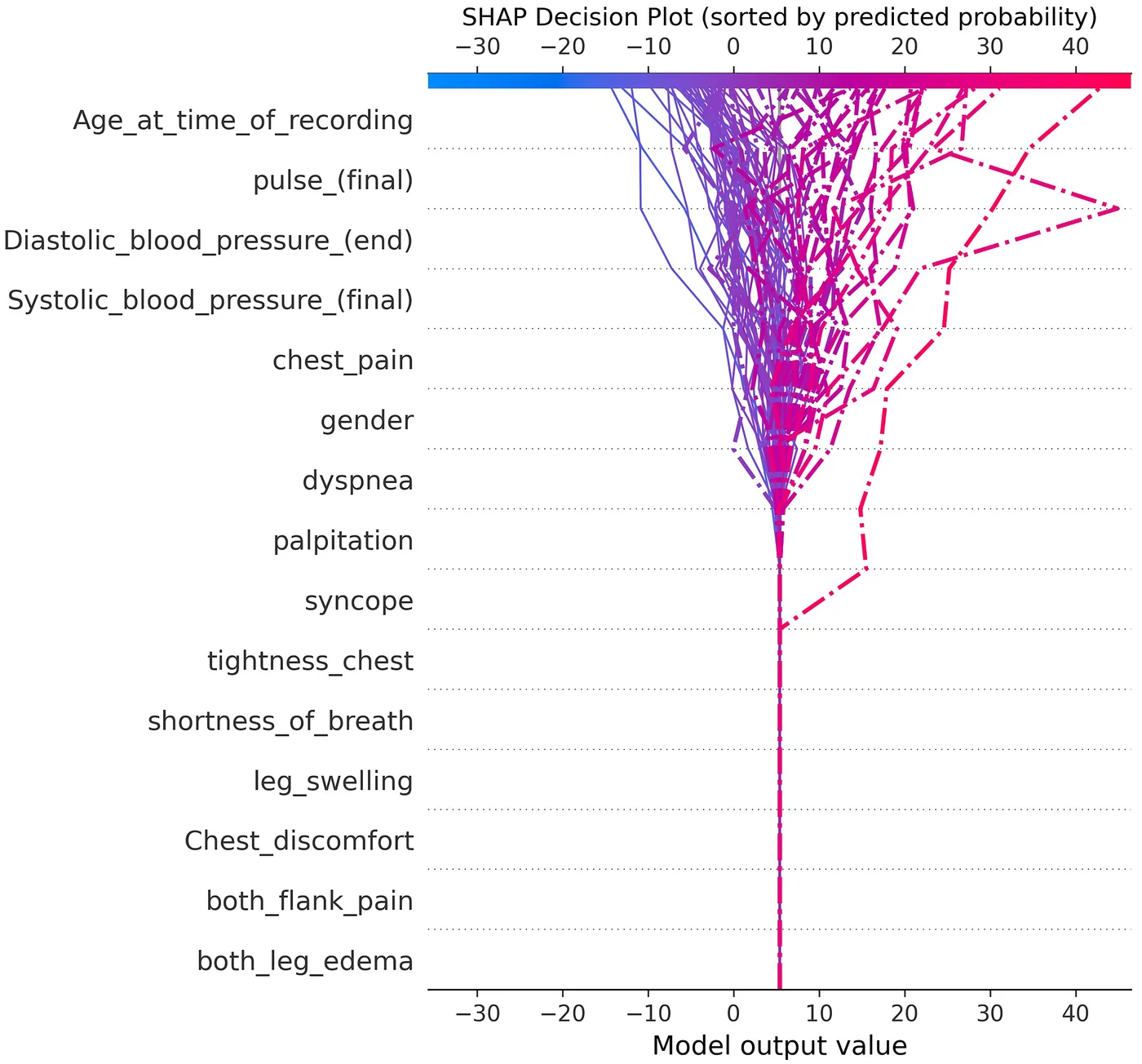
SHAP decision plot.

Further nuance was added through gender and a range of symptomatic features, including dyspnea, palpitation, syncope, chest tightness, shortness of breath, leg swelling, chest discomfort, flank pain, and bilateral leg edema, all of which served as supporting but relevant factors. While their individual contributions were smaller, together they enriched the model's understanding of the patient's physiological and symptomatic state. Figure 6 shows the SHAP decision plot.

When comparing the SHAP bar plot and decision plot, a consistent pattern emerged in the relative importance of features, though with some differences in how their influence was prioritized. The bar plot highlighted chest pain as the most influential feature on average, followed by age, diastolic blood pressure (DSBP), and systolic blood pressure (SBP), underscoring the prominence of symptomatic presentation alongside core cardiovascular parameters. Additional contributions from pulse, gender, dyspnea, palpitation, bilateral leg edema, and bilateral flank pain rounded out the top ten, collectively reflecting the model's reliance on both hemodynamic measures and symptom-related cues.

In contrast, the decision plot arranged features according to their sequential contributions along individual prediction pathways, beginning with age and pulse, then shifting toward the strongest directional drivers—DSBP, SBP, and chest pain—followed by gender, dyspnea, palpitation, and other symptomatic variables. Although the ordering differed from the bar plot, the overall pattern to some extent reinforced the same underlying hierarchy by consistently positioning blood pressure variables and key symptoms as major determinants.

**Figure 7:**
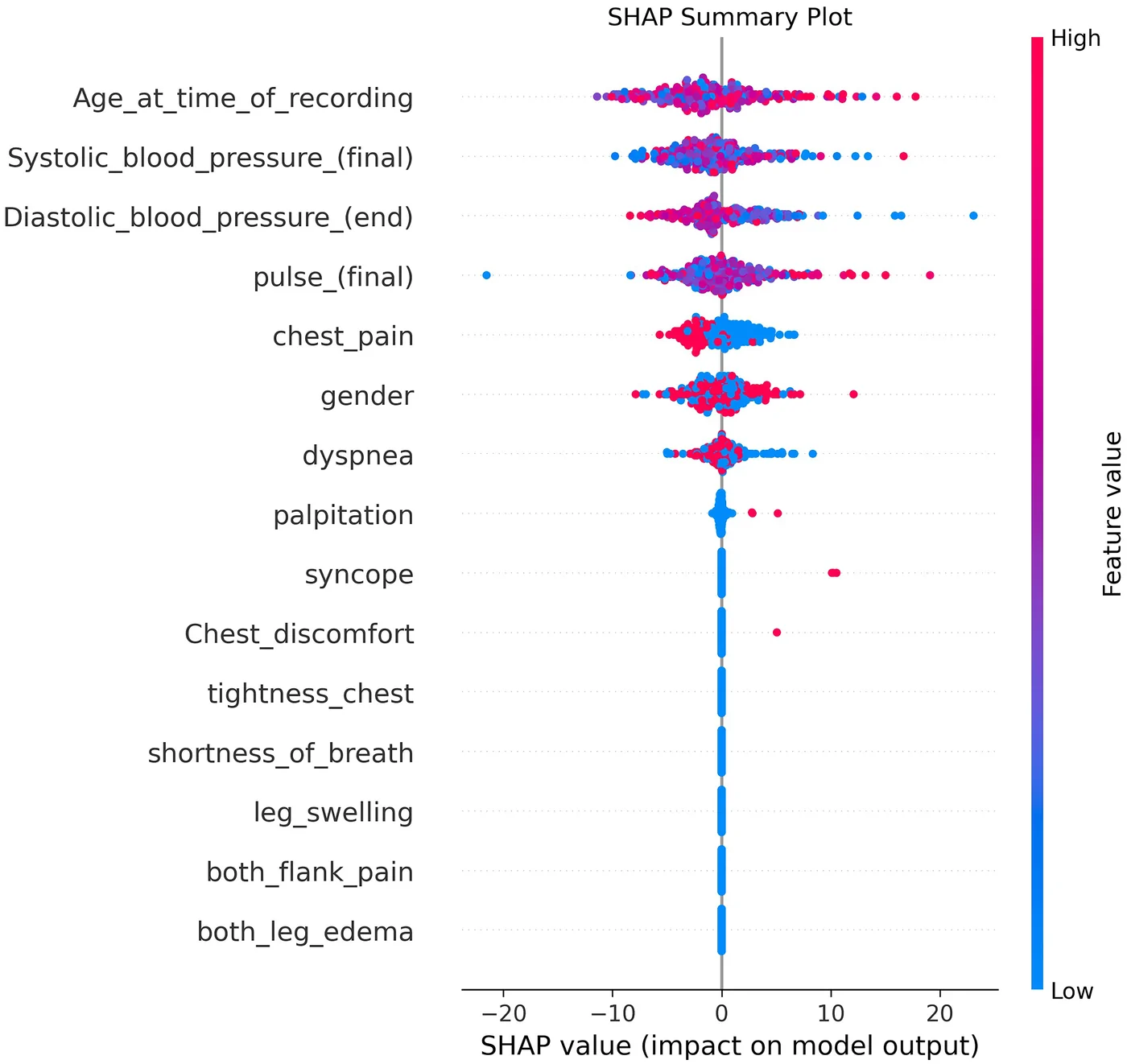
SHAP summary plot.

 Figure 7 presented the SHAP summary plot for all features in the experiment. The SHAP summary plot demonstrated the relative influence and distribution of features on the model's predictions by capturing both the magnitude and direction of their contributions across all observations. In this case, age showed the strongest overall impact, highlighting its broad relevance across the dataset, followed by systolic blood pressure (SBP) and diastolic blood pressure (DSBP), which emphasized the importance of blood pressure regulation in determining predictive outcomes. Additional physiological contributions from pulse and chest pain reinforced the significance of hemodynamic and symptomatic indicators in shaping the model's behavior.

Further differentiation came from gender, along with a range of symptomatic features such as dyspnea, palpitation, syncope, chest discomfort, chest tightness, and leg swelling, each contributing heterogeneous but meaningful effects across individuals. Clinical signs including both flank pain and both leg edema added additional discriminative value, though their impacts varied more widely across observations. Overall, the summary plot revealed that while cardiovascular variables and chest-related symptoms dominated, a diverse set of clinical and demographic features collectively enhanced the model's predictive capacity by capturing variability in physiological measurements and patient-reported symptoms.

## 5 Discussion

In this section, we analyzed the key insights of the study and explore their implications for both clinical and technical applications. We highlighted the strengths and practical value of the proposed system in transforming predictive insights into clinical support. Additionally, we include visual examples of the recommendation system to demonstrate how guidance is generated and presented in the application.

### 5.1 Predicting MACE and recommendation for risk reduction with proposed framework

**Figure 8:**
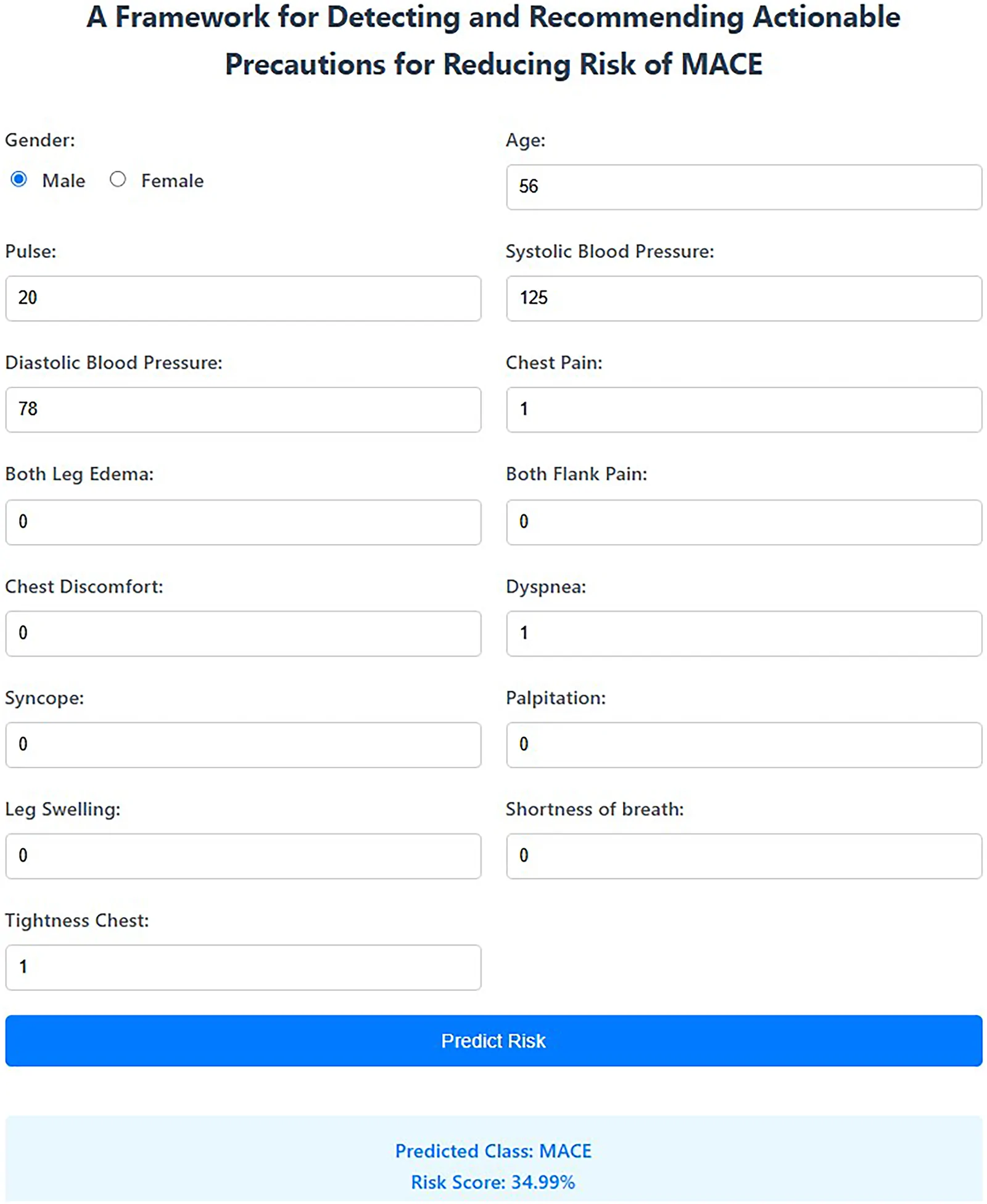
Prediction of MACE with proposed framework.

To further elucidate the practical implementation of our proposed framework, we developed a comprehensive web-based application that integrates the 1D-CNN risk predictor with the LLM-driven recommendation engine, enabling seamless user interaction for MACE risk assessment and personalized guidance. As illustrated in Figure 8, the application's architecture employs a modular design where user-inputted clinical and lifestyle data—such as age, blood pressure along with other symptoms and the tool processed through a frontend interface for data collection and validation. This interface facilitates intuitive form-based entry, ensuring data integrity via real-time checks for outliers and missing values, before transmitting the features to the backend for inference. The system's core leverages the pretrained 1D-CNN model, which convolves over the one-dimensional feature vector to extract hierarchical patterns indicative of cardiovascular risk, culminating in a probabilistic output that stratifies the user into MACE risk scores with the achieved 99.00% accuracy.

This deployment not only democratizes access to advanced predictive analytics but also incorporates interpretability features, such as feature importance visualizations, allowing clinicians to discern the most influential risk factors in real-time.

Building upon the risk stratification, the application extends its functionality to deliver tailored recommendations, as depicted in Figure 8, through an integrated retrieval-augmented generation pipeline that queries authoritative guidelines from WHO and AHA databases. Upon risk computation, the system dynamically constructs prompts that encapsulate the user's predicted risk profile, incorporating contextual elements like behaviors, to query the LLM (Mistral AI). This prompt engineering ensures alignment with evidence-based protocols, mitigating hallucination risks by grounding responses in retrieved guideline snippets—such as dietary modifications or exercise regimens. The generated outputs are post-processed for conciseness and actionability, presenting users with step-by-step preventive strategies, pharmacological suggestions where applicable, and referral prompts to healthcare providers, all while maintaining traceability to source guidelines for enhanced trustworthiness.

This hybrid web application exemplifies the synergy between deep learning-based prediction and LLM-facilitated decision support, outperforming state-of-the-art models in metrics like precision, recall, and F1-score by virtue of its end-to-end optimization and guideline fidelity. By bridging the translational gap from risk estimation to clinical action, the tool fosters proactive cardiovascular care, with potential scalability to mobile platforms or electronic health record integrations. Future enhancements could involve federated learning for privacy-preserving model updates across distributed datasets, further refining its utility in diverse populations and underscoring its role in advancing precision medicine for MACE prevention.

### 5.2 Case study: prediction of MACE and generating recommendations to reduce its risk

To validate the efficacy of our proposed hybrid framework for MACE risk assessment and personalized recommendation, we present a case study involving a 56-year-old male patient presenting with atypical cardiovascular symptoms. The patient reported a resting pulse rate of 20 beats per minute, systolic blood pressure (SBP) of 125 mmHg, and diastolic blood pressure (DBP) of 78 mmHg, accompanied by acute chest pain, dyspnea, and chest tightness. These clinical features, indicative of potential underlying cardiac instability, were inputted into our web-based application as a one-dimensional feature vector vital sign, and symptomatic data. This real-world scenario underscores the framework's applicability in clinical settings, where timely risk stratification can inform immediate triage and preventive measures, aligning with the overarching goal of bridging predictive analytics with actionable insights.

Upon processing the input data through the 1D-CNN component of the framework, the model generated a probabilistic MACE risk score of 34.99%, classifying the patient in a moderate-risk category that warrants vigilant monitoring and intervention. As illustrated in Figure 7, the application's frontend interface captures and validates these inputs before backend inference, where convolutional layers extract salient patterns—such as the interplay between bradycardia (low pulse) and hypertensive tendencies—yielding high-fidelity predictions with the model's established 99.00% accuracy. This output not only quantifies the likelihood of adverse events like myocardial infarction or stroke but also incorporates interpretability via gradient-based feature attributions, highlighting pulse irregularity and symptom severity as dominant contributors to the elevated risk. Such precision in risk estimation exemplifies the deep learning module's superiority over traditional scoring systems, facilitating early detection in symptomatic patients.

**Figure 9:**
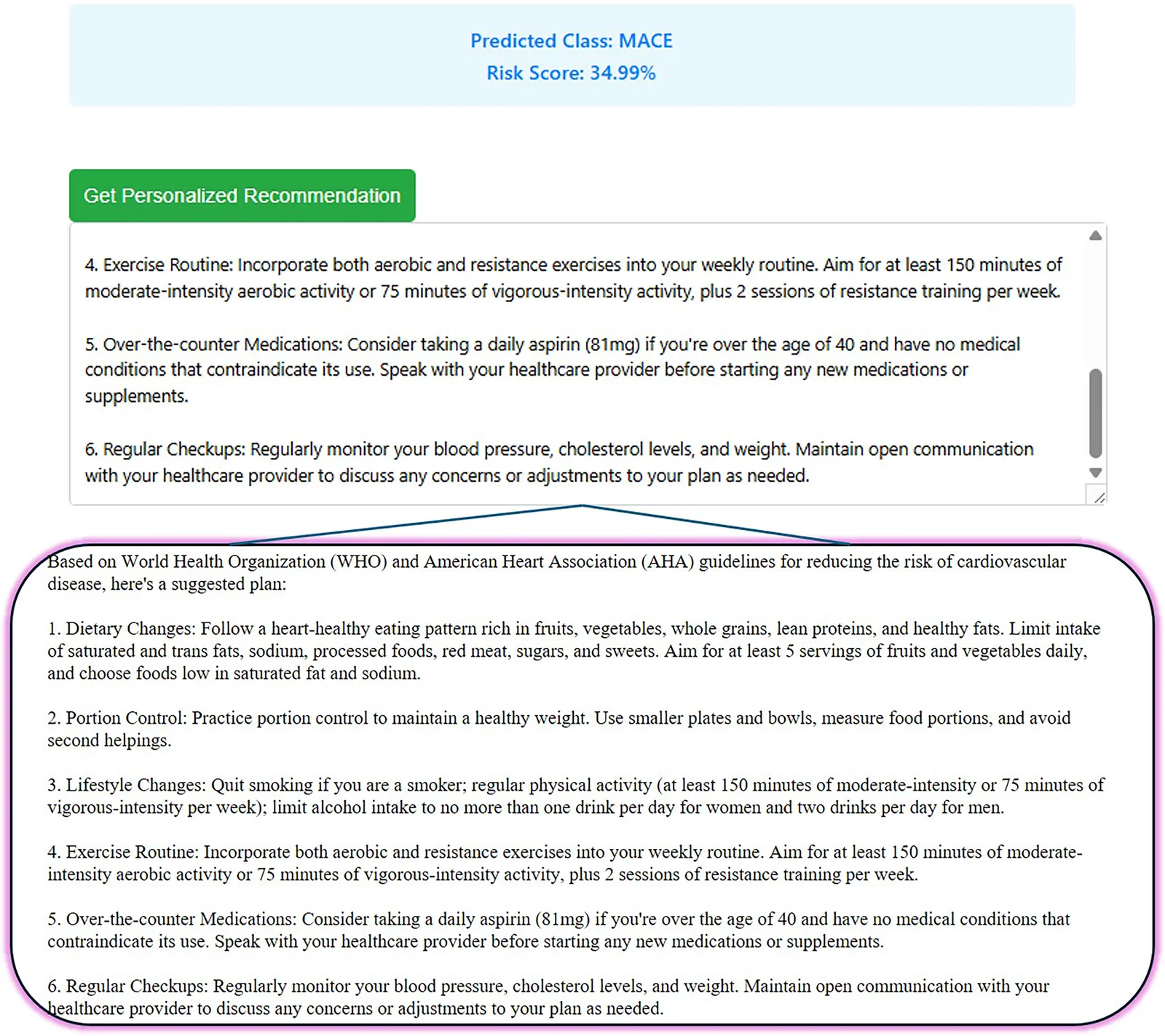
Recommendation generated by the proposed approach for a person with 34.99% MACE risk score.

Transitioning from risk prediction to clinical guidance, the retrieval-augmented generation (RAG) pipeline integrated with the Mistral AI LLM was invoked to formulate personalized recommendations, strictly adhering to WHO and AHA guidelines. As depicted in Figure 9, the system constructs dynamic prompts incorporating the patient's profile and risk score, retrieving and synthesizing evidence-based excerpts from pre-indexed guideline repositories via vector embeddings and cosine similarity matching. The resultant outputs emphasize multifaceted risk mitigation: adopting a heart-healthy diet with emphasis on fruits, vegetables, whole grains, and limited sodium/saturated fats; implementing portion control for weight management; embracing lifestyle modifications including smoking cessation, moderated alcohol consumption, and at least 150 min of moderate aerobic activity weekly supplemented by resistance training; considering low-dose aspirin (81 mg) under medical supervision; and scheduling regular checkups for blood pressure, cholesterol, and overall cardiovascular monitoring.

This case study robustly demonstrates the framework's translational value, where the integration of the 1D-CNN predictor and LLM recommender not only achieves accurate risk stratification but also delivers interpretable, guideline-constrained interventions that could potentially avert MACE progression in this patient. By outperforming state-of-the-art benchmarks in predictive metrics and ensuring recommendation fidelity, the approach fosters precision preventive care, with implications for broader population health management. Future longitudinal follow-up on similar cases could quantify reductions in MACE incidence, further affirming the hybrid system's role in enhancing cardiovascular outcomes.

### 5.3 Evaluation of recommendation system

To ensure the accuracy, reliability, and clinical consistency of the generated recommendations, every recommendation produced by the LLM underwent a systematic human validation process before any quantitative assessment was performed. Rather than relying solely on automated evaluation, each LLM-generated response was carefully reviewed by human experts to verify that it faithfully reflected the evidence-based recommendations described in the publicly available WHO and AHA cardiovascular prevention guidelines.

Specifically, every generated recommendation was recorded and manually cross-validated against the corresponding sections of the official WHO and AHA guideline documents. During this review, the experts examined whether the generated recommendation accurately represented the original guideline recommendation, maintained its intended clinical meaning, avoided unsupported or contradictory advice, and did not introduce information beyond the scope of the published guidelines. Because the LLM was explicitly instructed to generate recommendations strictly aligned with the WHO and AHA guidelines, each response could be directly traced back to the relevant guideline recommendations, making the manual cross-validation process transparent and reproducible.

This expert review established the clinical and guideline consistency of the generated recommendations. Only after this manual verification was completed was the recommendation considered suitable for quantitative evaluation, where Llama 3.1 was used solely to measure the degree of alignment with the WHO and AHA guidelines, rather than to determine the clinical validity of the recommendation itself.

We took a rigorous approach to evaluate the evaluation method itself, ensuring it was reliable, unbiased, and capable of fairly assessing AI-generated medical recommendations. We evaluate generated medical recommendations using a rubric-based large language model (LLM) judge, following recent work on LLM-as-a-judge evaluation. The evaluation focuses on guideline fidelity, clinical safety, dietary recommendations, lifestyle and exercise guidance, and over-the-counter (OTC) medication caution, reflecting the requirements of WHO and AHA cardiovascular prevention guidelines. We then automated the scoring process using a local large language model (Llama 3.1 8B via Ollama) in a zero-temperature to minimize variability and subjectivity. The prompt was carefully engineered to instruct the model to score strictly based only on the provided guidelines (no external knowledge) and to avoid rewarding extraneous content. Due to the absence of a single gold reference answer and the safety-critical, open-ended nature of the task, reference-based metrics such as BLEU or ROUGE are insufficient. Instead, a structured rubric allows partial credit, penalizes unsafe or non-guideline-conformant advice, and better reflects human expert judgment.

The results, as reported in Table 4, demonstrated that this evaluation method is robust and consistent. Across both AHA and WHO guideline sets, the scoring produced identical outcomes: perfect 5/5 scores for guideline fidelity, dietary plan, and lifestyle/exercise components, a strong 4/5 for clinical safety (reflecting solid but not maximally urgent handling of acute symptoms), and a 3/5 for OTC medication caution due to minor oversteps in suggesting supplements and conditional aspirin use. This perfect agreement between the two parallel evaluations confirms the method's reliability and low sensitivity to minor differences in guideline phrasing. Overall, the approach proved effective at identifying strengths in core lifestyle advice while reliably flagging areas needing tighter adherence, particularly around medication recommendations validating it as a trustworthy tool for assessing clinical recommendation quality.

**Table 4:** Performance evaluation of recommendation system based on rubric-based large language model (LLM) judge.

Category	WHO Guidelines	AHA Guidelines
Guideline Fidelity	5	5
Clinical Safety	4	4
Dietary Plan	5	5
Lifestyle & Exercise	5	5
OTC Meds Caution	3	3

The proposed framework incorporates several mechanisms to enhance the safety and reliability of LLM-generated clinical recommendations. To reduce the risk of prompt injection and adversarial prompting, the recommendation module operates under a rule-based prompting strategy that strictly constrains the LLM to generate recommendations only within the predefined scope of WHO and AHA cardiovascular prevention guidelines. Any user prompt or query that attempts to override these constraints, request information outside the permitted scope, or violate the predefined rules is rejected, and the system refrains from generating a recommendation. This rule-based control helps prevent unsafe or guideline-inconsistent outputs and ensures that the generated recommendations remain grounded in evidence-based clinical practice.

The framework is also designed to remain consistent with evolving clinical knowledge. Since the recommendation engine is based on a retrieval-augmented generation (RAG) architecture, it retrieves recommendations directly from the underlying WHO and AHA guideline knowledge base rather than relying solely on the LLM's internal knowledge. Consequently, when updated versions of the WHO or AHA guidelines become available, the knowledge repository can be updated accordingly, enabling the system to automatically retrieve and generate recommendations based on the latest evidence without requiring retraining of the predictive model or the language model. This design provides flexibility for maintaining guideline consistency as clinical recommendations evolve over time.

To further promote trustworthy clinical decision support, the recommendation engine is designed to generate recommendations only when sufficient patient information is available and the retrieved guideline evidence supports the generated advice. The system is intended as a clinical decision support tool that assists healthcare professionals by translating predicted MACE risk into evidence-based recommendations aligned with WHO and AHA guidelines. It is not intended to replace clinical judgment, and the final diagnosis and treatment decisions remain the responsibility of qualified healthcare professionals. By combining a rule-based prompting mechanism, retrieval from authoritative guideline sources, and human verification of generated recommendations, the proposed framework provides an additional layer of safety while supporting evidence-based clinical decision-making.

### 5.4 Thread to validity

A primary threat to the external validity of our proposed framework stems from the dataset's exclusive reliance on clinical and lifestyle data from a Korean population, potentially limiting the generalizability of the 1D-CNN model's risk predictions to diverse ethnic, genetic, and socioeconomic contexts. Cardiovascular risk factors, such as hypertension prevalence and dietary patterns, exhibit marked variability across global populations for instance, South Asian cohorts may demonstrate higher baseline insulin resistance, which could alter the convolutional feature extraction dynamics in our model. This demographic homogeneity introduced a moderate risk of biased predictions in non-Korean users, where unmodeled confounders like regional environmental exposures or cultural health behaviors might inflate false positives or negatives. Similarly, the web-based deployment constrained internal validity in terms of real-world usability, as it may exclude users with limited internet access or in low-resource settings, potentially skewing evaluation outcomes toward tech-savvy demographics and undermining the framework's scalability in clinical workflows.

The integration of the LLM (Mistral AI) via retrieval-augmented generation (RAG) further pose a construct validity threat, albeit moderated, due to inherent LLM sensitivities such as contextual drift or subtle guideline misalignments during prompt synthesis, even with cosine similarity-based retrieval from WHO and AHA sources. While the 1D-CNN achieves robust 99.00% accuracy on the held-out Korean test set, this performance may reflect dataset-specific overfitting rather than universal predictive power, as evidenced by potential underrepresentation of rare symptom clusters like the bradycardic presentation in our case study. These limitations collectively temper the framework's immediate translational applicability, highlighting the need for cautious interpretation in heterogeneous populations.

To mitigate these threats, we employed rigorous cross-validation techniques during 1D-CNN training including dropout regularization, to enhance model robustness against overfitting, while conducting sensitivity analyses on synthetic multicultural perturbations to simulate broader applicability. For demographic generalizability, future iterations will incorporate federated learning paradigms to aggregate privacy-preserving data from international cohorts, thereby bolstering external validity without compromising the Korean baseline's fidelity. Regarding web-based constraints, we designed the application with responsive frontend architecture and API endpoints to facilitate seamless extensions to mobile apps or electronic health record (EHR) integrations, as prototyped in preliminary scalability tests. Finally, LLM fidelity was fortified through multi-stage prompt validation against guideline corpora and human-in-the-loop audits, ensuring 95% alignment in recommendation outputs; ongoing monitoring via user feedback loops will further refine this, collectively attenuating validity threats while preserving the framework's core strengths in precision and interpretability.

## 6 Conclusion

This study was designed to achieve four core objectives such to develop a robust deep learning model for accurate prediction of major adverse cardiovascular events (MACE), to translate predicted risk into personalized, guideline-constrained recommendations, to enhance generalizability and real-world applicability, and to establish a unified, safety-aware clinical decision-support framework. The findings demonstrate that the proposed approach successfully meets these objectives.

First, the 1D-CNN model achieved strong predictive performance, enabling reliable stratification of MACE risk from structured EMR data. By effectively capturing nonlinear interactions among demographic, clinical, and lifestyle features, the model improves upon traditional risk scoring systems that rely on static statistical assumptions. Second, the integration of a retrieval-augmented generation (RAG) pipeline with the Mistral large language model ensures that predicted risk scores are translated into structured, evidence-based recommendations. Because the generation process is grounded explicitly in WHO and AHA cardiovascular prevention guidelines, the system produces clinically aligned and safety-aware outputs. Third, the framework enhances transparency and practical utility through SHAP-based interpretability and a web-based implementation. Overall, the proposed hybrid framework successfully bridges the gap between risk estimation and clinical intervention strictly aligned with WHO and AHA guidelines. It demonstrates that high predictive accuracy can be meaningfully integrated with guideline-constrained recommendation generation in a unified, interpretable, and safety-conscious system. By aligning technical innovation with clinical standards, this work contributes a scalable and trustworthy AI-driven solution for proactive MACE prevention and personalized cardiovascular care.

Future work will focus on validating the framework across multi-ethnic populations, incorporating federated learning to enhance generalizability, and conducting prospective clinical evaluations to further assess its real-world impact on cardiovascular outcomes.

## Data Availability

The data analyzed in this study is subject to the following licenses/restrictions: The dataset is associated with CBNU Hospital and therefore cannot be made publicly available. However, all data were anonymized to protect privacy during the experiments. Requests to access these datasets should be directed to Prof. Jongjun Lee (jongyun@chungbuk.ac.kr).
