# Correction: A hybrid deep learning and large language model framework for MACE risk prediction and evidence-based clinical recommendations

**DOI:** 10.3389/fcvm.2026.1967558

**Published:** 2026-08-31

**Authors:** Saba Arif, Sang Hyeok Son, Jong Yun Lee

**Affiliations:** Department of Computer Science, Chungbuk National University, Cheongju, Republic of Korea

**Keywords:** cardiovascular risk prediction, deep learning, health recommendation guideline, large language models (LLM), MACE

Grant/funding/project number incorrect

An incorrect project number was provided in the funding statement for the Regional Innovative System & Education (RISE) program. The published project number was 2026-RISE-11-014-01, and the correct project number is 2026-RISE-11-014-03. The remainder of the funding statement remains unchanged.

The original version of this article has been updated.

